# Histological Remodeling of Irradiated Postmastectomy Breast Tissue After Autologous Fat Grafting: A Prospective Paired Tru-Cut Biopsy Study

**DOI:** 10.3390/medsci14020180

**Published:** 2026-04-02

**Authors:** Razvan George Bogdan, Alina Helgiu, Anca Maria Cimpean, Mara Nicolau, Rodica Elena Heredea, Zorin Petrisor Crainiceanu

**Affiliations:** 1 Doctoral School, “Victor Babeș” University of Medicine and Pharmacy, 300041 Timisoara, Romania; razvan.bogdan@umft.ro; 2 County Clinical Emergency Hospital “Pius Brînzeu”, 300723 Timisoara, Romania; 3 Faculty of Medicine, “Lucian Blaga” University of Sibiu, 550024 Sibiu, Romania; maranicolau16@gmail.com; 4 County Clinical Emergency Hospital of Sibiu, 550245 Sibiu, Romania; 5 Department of Microscopic Morphology/Histology, “Victor Babeș” University of Medicine and Pharmacy, 300041 Timisoara, Romania; acimpeanu@umft.ro; 6 Center of Expertise for Rare Vascular Disease in Children, Emergency Hospital for Children Louis Turcanu, 300011 Timisoara, Romania; 7 Center of Genomic Medicine, “Victor Babeș” University of Medicine and Pharmacy, 300041 Timisoara, Romania; 8 Research Center for Pharmaco-Toxicological Evaluation, “Victor Babeș” University of Medicine and Pharmacy, 300041 Timisoara, Romania; 9 Discipline of Clinical Practical Skills, Department I Nursing, Faculty of Medicine, “Victor Babeș” University of Medicine and Pharmacy, 300041 Timisoara, Romania; 10 Plastic Surgery Department, “Victor Babeș” University of Medicine and Pharmacy, 300041 Timisoara, Romania

**Keywords:** autologous fat grafting, lipofilling, radiation-induced fibrosis, breast reconstruction, histological remodeling, irradiated tissue, connective tissue reorganization, Tru-Cut biopsy, postmastectomy reconstruction

## Abstract

**Background/Objectives**: Radiotherapy following mastectomy induces persistent structural alterations in the chest wall, including fibrosis, extracellular matrix disorganization, and vascular changes that compromise reconstructive outcomes. Although autologous fat grafting is widely used to improve tissue quality in irradiated breasts, direct human histological evidence remains limited. The aim of this prospective pilot study was to evaluate intra-patient histological remodeling in irradiated postmastectomy breast tissue before and 4 months after autologous fat grafting using paired core needle biopsies. This study should be considered a hypothesis-generating histological pilot study. **Methods**: Five female patients with prior mastectomy and adjuvant radiotherapy underwent Tru-Cut core needle biopsy of irradiated chest wall tissue before lipofilling and at approximately four months (range between 3 and 12 months) post-procedure. Specimens were processed using formalin fixation, paraffin embedding, and hematoxylin and eosin staining. Histological assessment focused on collagen density, stromal organization, vascular structures, inflammatory infiltrate, and adipocyte integration. Comparative intra-patient analysis was performed descriptively. **Results**: Baseline biopsies demonstrated consistent post-radiation alterations, including collagen compaction, stromal disorganization, perivascular fibrosis, and variable inflammatory infiltrate. Post-lipofilling specimens showed heterogeneous remodeling characterized by focal collagen fiber insertion between adipocytes, areas of immature connective tissue formation, and variable preservation of adipose architecture. The extent and pattern of remodeling differed among patients. Inflammatory activity decreased or remained mild in most cases. **Conclusions**: Autologous fat grafting in irradiated postmastectomy tissue is associated with measurable histological remodeling. Structural adaptation appears heterogeneous and patient-specific, suggesting a dynamic multi-stage process rather than uniform regeneration. Further studies incorporating quantitative and molecular analyses are required to clarify the mechanisms underlying these changes.

## 1. Introduction

Breast cancer remains the most frequently diagnosed malignancy among women worldwide, and mastectomy followed by adjuvant radiotherapy is a standard therapeutic approach in locally advanced disease and high-risk cases [1]. Although radiotherapy improves local control and survival, it induces long-term structural alterations in the irradiated chest wall. These changes include progressive fibrosis, extracellular matrix disorganization, vascular rarefaction, and chronic inflammatory remodeling [2,3].

Radiation-induced fibrosis represents a persistent and often irreversible process characterized by excessive collagen deposition, stromal hyalinization, and reduced tissue elasticity [4]. Clinically, patients present with skin retraction, reduced compliance, impaired wound healing, and compromised reconstructive outcomes. The irradiated microenvironment exhibits decreased vascular density and altered tissue architecture, which negatively impact surgical interventions performed in the affected area [5].

Autologous fat grafting has emerged as a reconstructive technique aimed not only at volume restoration but also at improving the quality of irradiated soft tissues [6]. Several clinical studies have reported improved skin pliability, reduced fibrosis, and better esthetic outcomes after lipofilling in previously irradiated breasts [7]. Experimental data suggest that adipose tissue may exert regenerative effects through paracrine signaling, modulation of inflammation, and promotion of neovascularization [8].

Despite these observations, direct histological evidence in human subjects remains limited. Most available data rely on clinical assessment, imaging findings, or experimental animal models rather than intra-patient structural comparison. Human studies rarely incorporate protocol-driven paired tissue sampling before and after fat grafting, particularly using standardized core needle biopsy protocols. As a result, structural changes at the microscopic level remain incompletely characterized.

Controversy persists regarding the extent to which autologous fat grafting can truly reverse radiation-induced fibrosis versus merely improving tissue compliance through volumetric augmentation [9]. A prospective paired intra-patient biopsy approach offers a methodological advantage by minimizing inter-individual variability and enabling direct structural comparison within the same biological environment.

The present prospective pilot study was designed from the outset to include protocol-driven paired intra-patient core needle biopsies in order to evaluate histological remodeling in irradiated postmastectomy breast tissue before and approximately four months after autologous fat grafting using paired Tru-Cut core needle biopsies. We hypothesized that fat grafting would be associated with measurable structural remodeling, reflected by reduced collagen compaction, improved stromal organization, and evidence of adipose tissue integration within the irradiated recipient bed.

To our knowledge, prospective intra-patient paired histological analysis using core needle biopsies in irradiated postmastectomy tissues remains rarely reported in human clinical studies. This design allows direct structural comparison within the same biological environment while minimizing inter-individual variability. Given the exploratory nature and limited sample size, this study was designed as a hypothesis-generating histological pilot investigation.

## 2. Materials and Methods

### 2.1. Study Design and Patient Selection

The present study reports the histological component of a prospective cohort that was designed from the outset to include both ultrasound-based and histological evaluation of structural changes following autologous fat grafting [10]. The prior publication focused exclusively on imaging outcomes, whereas the current study analyzes histological remodeling using paired Tru-Cut core needle biopsies. This was a prospective single-center hypothesis-generating pilot study with a longitudinal intra-patient comparative design.

This was a prospective single-center pilot study conducted between October 2023 and June 2024, evaluating histological changes following autologous fat grafting in irradiated chest wall tissues postmastectomy with a longitudinal intra-patient comparative design. Five female patients were recruited from the Department of Plastic and Reconstructive Surgery at “Victor Babes” University of Medicine and Pharmacy, Timisoara, Romania.

All patients completed the planned follow-up protocol, and no patient was lost to follow-up. Clinical checks took place at 2 weeks and 1 month, but these were not included in the histological analysis.

Inclusion criteria

Inclusion criteria were female sex, age ≥ 18 years, prior radical mastectomy followed by adjuvant external beam chest wall radiotherapy, completion of radiotherapy at least 6 months before enrollment, absence of local recurrence or metastatic disease, presence of clinical or structural signs of radiation-induced soft tissue damage such as subcutaneous atrophy or fibrotic scarring, and indication for autologous fat grafting as part of a reconstructive strategy.

Exclusion criteria

Exclusion criteria included uncontrolled systemic illness such as diabetes or cardio-vascular disease, active infection or inflammatory skin disease at the donor or recipient site, prior lipofilling in the same anatomical area, and anticoagulant therapy contraindicating fat grafting.

The demographic, oncological, and procedural characteristics of the included patients are summarized in Table 1 and Table 2.

### 2.2. Anesthesia and Perioperative Care

All procedures were performed under general anesthesia using intravenous sedation. Perioperative antibiotic prophylaxis was administered to all patients according to local hospital protocol. Postoperative thromboprophylaxis consisted of subcutaneous enoxaparin (Clexane^®^ Sanofi, Paris, France) 0.4 mL daily for 5 consecutive days, administered according to institutional standard prophylactic protocols for low-risk elective reconstructive procedures.

Patients were encouraged to mobilize on the same day after surgery as part of the standard thromboprophylaxis strategy. No thromboembolic events were recorded during the follow-up period.

### 2.3. Fat Harvesting and Processing

Donor areas included the lower abdomen, flanks, or medial thighs, selected based on adipose tissue availability and patient preference. Klein’s super-wet infiltration technique was employed, with tumescent solution injected and allowed to act for approximately 20 min prior to aspiration. Fat was harvested using a 4 mm blunt-tip cannula connected to a vacuum-assisted aspiration system with a sterile collection canister, which enabled the collection of lipoaspirate under continuous, controlled moderate negative pressure.

The aspirated fat was manually washed in a sterile container using five sequential rinses with 250 mL of sterile 0.9% saline solution. After each rinse, the lipoaspirate was allowed to decant for approximately 2 min. The middle purified layer was carefully collected for grafting, while the oil supernatant and erythrocyte-rich infranatant were discarded.

This technique was selected for procedural standardization, and the study was not designed to compare harvest efficiency or graft quality with other infiltration approaches.

### 2.4. Fat Injection Technique

Fat grafting was performed using a Coleman-type technique. Multiple small aliquots were injected in a retrograde fashion into multiple planes (superficial subcutaneous, deep subcutaneous, and beneath the mastectomy scar) using 50 mL Luer-lock syringes. Care was taken to avoid overcorrection and ensure uniform distribution. The average grafted volume per patient ranged between 130 and 170 mL per breast, depending on tissue capacity and clinical objectives.

No cell-assisted lipotransfer, ADSC isolation, or stromal vascular fraction enrichment was performed. This study used standard autologous fat grafting only.

### 2.5. Semi-Quantitative Histological Scoring System

To improve inter-case comparability and ensure structured reporting, a semi-quantitative descriptive grading system was applied to all predefined histological parameters. The grading scale was derived directly from the terminology used in the original histopathological evaluation.

Descriptive terms such as “absent” or “without” were coded as 0 (none). Terms including “slight,” “discrete,” “rare,” or “mild” were coded as 1 (mild). Findings described as “moderate” were coded as 2 (moderate). Descriptors such as “marked,” “intense,” “abundant,” “increased,” or “hypervascularization” were coded as 3 (marked).

This approach did not introduce new interpretative morphological criteria but translated the original descriptive terminology into a standardized four-tier ordinal scale to allow structured intra-patient comparison between baseline and post-lipofilling specimens.

The following parameters were graded:

1. Fibrosis/Dense Disorganized Connective Tissue

Parameter: Degree of collagen deposition and structural replacement by dense disorganized connective tissue.

0 (None): No increase in collagen deposition; preserved adipose architecture.

1 (Mild): Slight or discrete collagen fibers; rare or thin collagen strands without architectural distortion.

2 (Moderate): Clearly identifiable collagen bundles or reticular collagen networks, without complete replacement of adipose structure.

3 (Marked): Abundant collagen deposition with dense disorganized connective tissue demonstrating fascicular organization and structural predominance over adipose tissue.

2. Inflammatory Infiltrate

Parameter: Degree of inflammatory cell presence within adipose and stromal compartments.

0 (None): No inflammatory infiltrate.

1 (Mild): Sparse or focal inflammatory cells without architectural impact.

2 (Moderate): Moderate inflammatory cell presence distributed within adipose or stromal areas.

3 (Marked): Intense or abundant inflammatory infiltrate involving multiple fields.

3. Vascular Alterations

Parameter: Degree of vascular density increase and microvascular structural alterations.

0 (None): No vascular proliferation or abnormal vascular features.

1 (Mild): Slight increase in small-caliber vessels without architectural distortion.

2 (Moderate): Clearly increased vascular density compared to adjacent tissue fields.

3 (Marked): Hypervascularization with abundant vascular profiles or prominent vascular proliferation.

4. Perivascular Connective Tissue Thickening

Parameter: Extent of concentric collagen deposition surrounding small vascular structures.

0 (None): No perivascular collagen thickening.

1 (Mild): Subtle or focal perivascular collagen deposition.

2 (Moderate): Evident perivascular thickening in multiple microscopic fields.

3 (Marked): Prominent concentric collagen deposition surrounding vessels with clear structural thickening.

This semi-quantitative scoring system is exploratory in nature and was derived from descriptive histopathological terminology without prior validation. The assessment is observer-dependent and may be subject to interpretation variability.

### 2.6. Histological Evaluation Protocol

Core needle biopsies were obtained intraoperatively using a 14-gauge Tru-Cut needle from irradiated chest wall tissue immediately prior to autologous fat grafting. A second intraoperative biopsy was performed approximately four months later during the subsequent reconstructive procedure (repeat fat grafting, implant-based reconstruction, expander placement, or autologous tissue reconstruction such as DIEP or latissimus dorsi flap). Sampling targeted the same anatomical region and anatomically comparable tissue planes within the irradiated reconstruction area to ensure spatial and procedural consistency between paired specimens. One representative core specimen was collected at each timepoint for each patient.

Specimens were immediately fixed in 10% neutral buffered formalin for 24 h, followed by routine paraffin embedding. Tissue blocks were sectioned at 4 µm thickness using a rotary microtome and mounted on glass slides. Sections were stained with hematoxylin and eosin according to standard histopathological laboratory protocols.

Histological evaluation was performed using light microscopy at magnifications ranging from ×40 to ×100. Assessment focused on collagen density and compaction, stromal architectural organization, presence and distribution of vascular structures, inflammatory cell infiltrate, and evidence of adipocyte survival and integration within the recipient bed.

Fibrosis, vascularity, inflammatory infiltrate, and stromal organization were assessed using a semi-quantitative ordinal grading system based on predefined morphological criteria. Fibrosis was graded according to collagen bundle thickness and stromal compaction. Vascularity was evaluated based on vessel density and morphological characteristics. Inflammatory infiltrate was categorized as absent, mild, moderate, or prominent. All parameters were evaluated consistently across paired specimens to allow structured intra-patient comparison.

Paired baseline and post-treatment specimens were comparatively analyzed to identify structural changes associated with autologous fat grafting. All histological assessments were performed by an experienced pathologist who was aware of the temporal sequence of the specimens but applied predefined morphological criteria for fibrosis, vascularity, inflammatory infiltrate, and stromal organization to ensure standardized evaluation across paired samples.

Digital color segmentation analysis was performed using RGB channel separation to differentiate collagen fibers from adipose structures.

For each biopsy specimen, at least five representative microscopic fields were evaluated.

Adipocyte integrity was assessed morphologically based on preservation of cell membrane contour, cytoplasmic vacuolar architecture, and absence of nuclear fragmentation or cellular debris.

### 2.7. Data Collection and Analysis

Histological findings were recorded for each patient at two timepoints, baseline and four months after autologous fat grafting. Data collection focused on predefined morphological parameters aimed at characterizing structural remodeling, primarily defined by intra-patient changes in collagen organization and stromal architecture, together with modulation of inflammatory infiltrate, vascular patterns, and adipocyte integration.

Comparative intra-patient analysis was performed between paired baseline and post-treatment specimens. Structural changes were documented descriptively, with emphasis on differences in collagen compaction, degree of stromal hyalinization, architectural organization, and relative vascular presence.

Given the exploratory design and limited sample size of five patients, data interpretation was restricted to descriptive comparative assessment. Findings were analyzed within each subject to identify consistent patterns of tissue remodeling following autologous fat grafting.

Statistical Analysis

In line with the exploratory pilot design and limited cohort size, analyses were confined to structured descriptive intra-patient comparisons, and no inferential statistical testing was undertaken. Data were summarized descriptively.

### 2.8. Ethical Considerations

All procedures complied with the ethical principles of the Declaration of Helsinki.

Patients were fully informed regarding the nature, objectives, risks, and potential benefits of the study. Written informed consent was obtained prior to inclusion. Study approval was granted by the Institutional Ethics Committee of “Victor Babes” University of Medicine and Pharmacy, Timisoara (Approval No. 54/25, date 25 November 2022 revised in 20 February 2026).

## 3. Results

### 3.1. Baseline Histological Findings (Pre-Lipofilling)

All five patients demonstrated consistent post-radiation structural alterations in the pre-lipofilling biopsies. Common baseline findings included:

- Condensation and compaction of collagen fibers with areas of dense disorganized connective tissue;
- Perivascular collagen thickening and stromal hyalinization;
- Increased extracellular matrix deposition;
- Variable degrees of inflammatory infiltrate;
- Vascular alterations, including increased capillary density in selected cases.

In several specimens, adipose tissue adjacent to fibrotic areas appeared relatively preserved, while dense connective tissue showed architectural distortion and collagen bundle disorganization. In two cases, isolated adipocytes were observed within dense disorganized connective tissue. One case exhibited vascular lacunae containing erythrocytes without clearly identifiable endothelial lining. Moderate inflammatory infiltrate with macrophage presence was observed in selected specimens. Clear demarcation between fibrotic areas and preserved adipose tissue was identifiable in most baseline samples.

### 3.2. Post-Lipofilling Histological Changes

At three to six months following autologous fat grafting, all patients demonstrated structural remodeling of the treated area. However, the pattern and extent of remodeling varied between individuals.

General post-treatment findings included:

- Reduction in organized adipocyte clusters in selected regions;
- Presence of newly formed collagen fibers interspersed between adipocytes;
- Areas of immature connective tissue characterized by thin collagen fibers;
- Focal organization of collagen bundles into short fascicular arrangements;
- Heterogeneous distribution of adipose preservation and fibrotic transformation.

In multiple cases, adipocytes appeared reduced in size with partial architectural disruption. In some regions, adipose tissue alternated with areas of dense disorganized connective tissue, suggesting progressive remodeling. Post-treatment inflammatory infiltrate was mild or absent in four of five patients, with one patient demonstrating persistent inflammatory activity. Vascular density remained increased in a subset of patients, while partial normalization compared to baseline was observed in others, indicating heterogeneous vascular remodeling.

The interval between baseline and post-lipofilling biopsy ranged from 3 to 12 months. Early post-treatment specimens obtained at approximately 3–4.5 months already demonstrated active structural remodeling characterized by collagen insertion and stromal reorganization. In the single case evaluated at 12 months, areas of more organized collagen bundles and advanced connective tissue structuring were observed, suggesting temporal maturation of the remodeling process. Qualitative assessment revealed variable proportions between residual adipose tissue and areas of dense disorganized connective tissue across cases, with the relative balance between these compartments differing among patients.

### 3.3. Individual Case Characteristics

#### 3.3.1. Patient 1 (Figure 1, Figure 2 and Figure 3)

Pre-treatment biopsy showed marked inflammatory infiltrate, perivascular collagen thickening, and hypervascularization of adipose tissue. Post-treatment specimens demonstrated persistent inflammatory activity and maintained vascular density. Collagen deposition between adipocytes was present but limited, and overall adipose architecture remained partially preserved.

**Figure 1:**
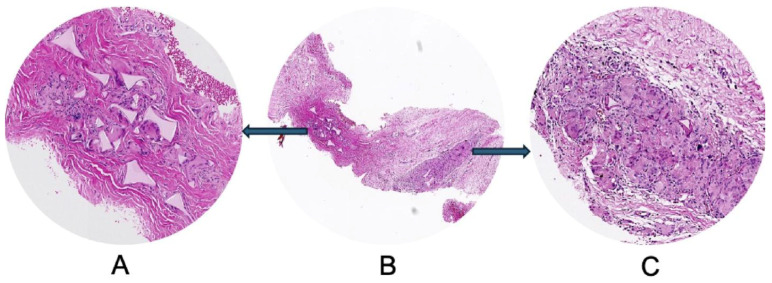
Histopathological evaluation of patient P1 before lipofilling. Hematoxylin and eosin staining (H & E). (**A**) Dense fibrotic stroma of the breast tissue, characterized by thick collagen bundles and marked stromal sclerosis, consistent with chronic post-procedural remodeling. (**B**) Tissue fragment containing multiple optically empty spaces compatible with foreign material residues from previous procedures, surrounded by fibrous tissue reaction. (**C**) Hypercellular fibrous tissue associated with a prominent inflammatory infiltrate composed predominantly of lymphocytes and macrophages, indicating a chronic inflammatory response likely related to persistent foreign material fragments, possibly silicone.

**Figure 2:**
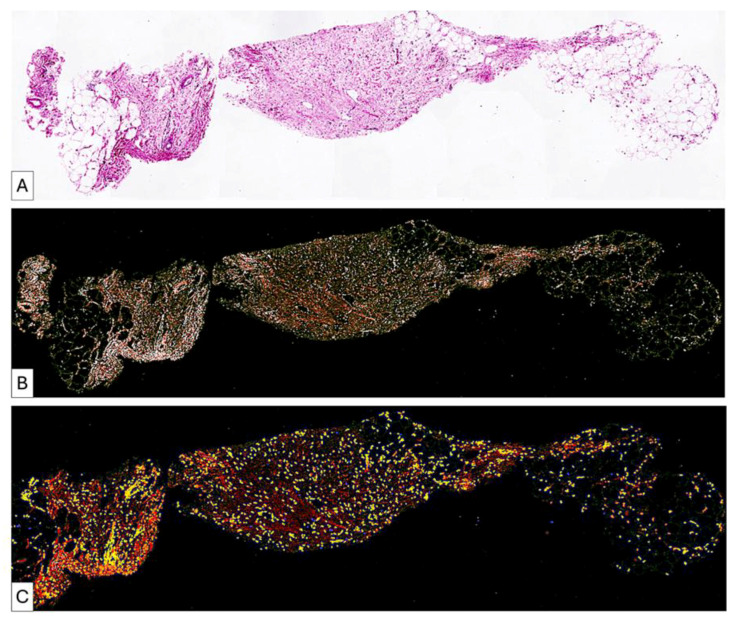
Three months post lipofilling, another tissue biopsy was harvested. Unfortunately for the patient, adipose tissue used for lipofilling was found to be invaded by lymphocytes (**A**) as newly formed connective tissue was, also. Collagen fibers derived by adipose tissue remodeling were thin and sparse with low ability of lateral aggregation to form fascicles. Despite of long time between initial implant and tissue biopsy, we observed the persistence of green fibrillar components as fragments of the previous adipose cells intermixed with fine collagen fibrils not organized in fascicles. They form a loose connective tissue (**B**) highly cellular (**C**).

**Figure 3:**
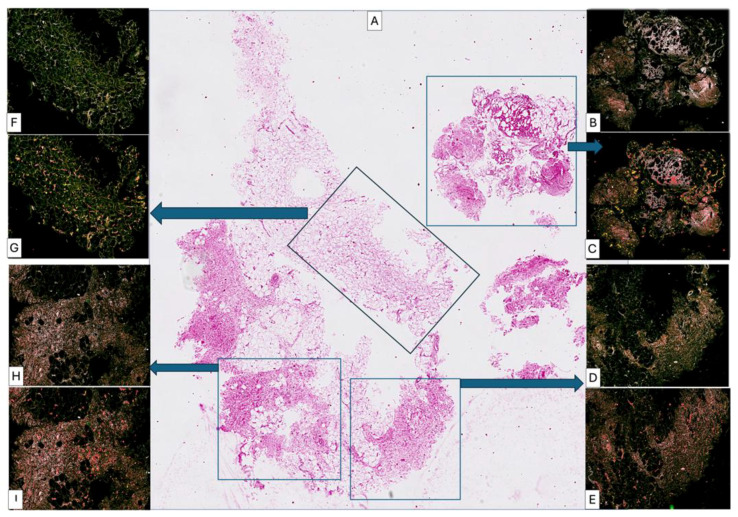
Biopsy taken after second lipofilling was composed of highly heterogeneous areas intermixing adipose tissue with no signs of differentiation through a connective tissue (middle quadrant (**A**,**F**,**G**)) and well differentiated compact areas of connective tissues ((**A**), lower quadrant, (**D**,**E**,**H**,**I**)) but still infiltrated with high amounts of lymphocytes. Although the inflammatory process still persist, collagen fibrils were aggregated to form collagen fascicles arranged in different directions (one of the main criteria of dense irregular connective tissue). Some areas were already composed of well-organized dense irregular connective tissue ((**A**), upper right quadrant, (**B**,**C**)) but with persistence of partial differentiation of adipose tissue and low cellular components.

#### 3.3.2. Patient 2 (Figure 4)

Baseline tissue demonstrated disorganized connective tissue with moderate inflammatory infiltrate and crown-like adipocyte structures. Post-lipofilling biopsy revealed morphologically altered adipose tissue with persistent capillary vascularity and minimal collagen fiber formation. Connective tissue appeared more lax and less densely organized compared to baseline.

**Figure 4:**
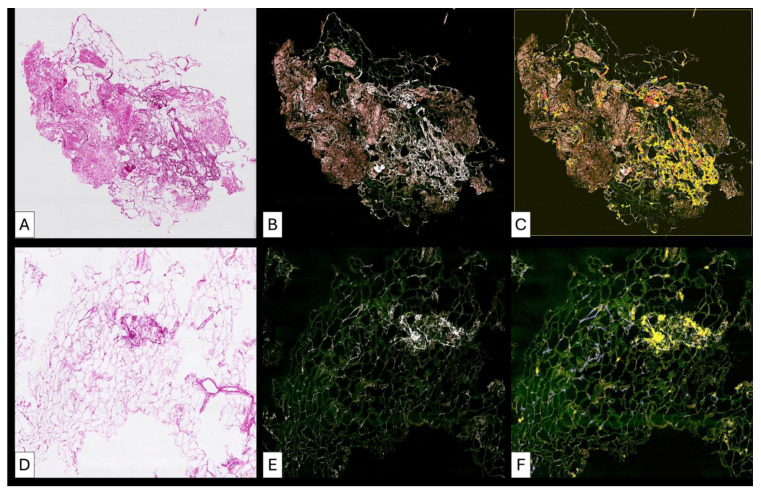
High heterogeneity of adipose tissue differentiation after lipofilling. The pre lipofilling tissue (**A**) includes area of mature fibrosis intermixed with areas of immature collagen fibrils (**B**) and high cellular content (**C**). Post lipofilling the same pattern persists. Adipose tissue used for lipofilling (**D**) had a higher ability to transdifferentiate into fibrous tissue (**E**) by a cellular mediated remodelingprocess (**F**).

#### 3.3.3. Patient 3 (Figure 5, Figure 6 and Figure 7)

Pre-lipofilling biopsy showed vascular lacunae with abundant erythrocytes and isolated adipocytes within dense connective tissue. Post-treatment specimens demonstrated insertion of thin collagen fibers between adipocytes, focal adipocyte regression, and early fascicular organization of collagen bundles. Remodeling was heterogeneous with areas of preserved adipose tissue alternating with fibrotic transformation.

**Figure 5:**
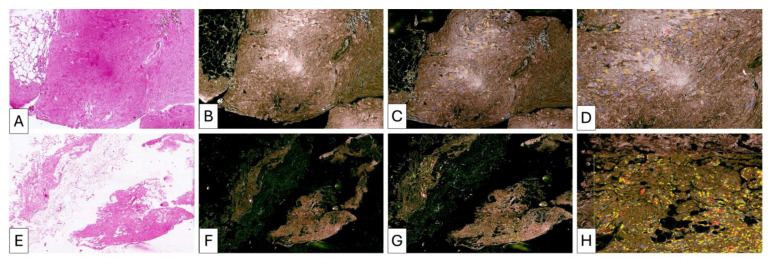
(**A**–**H**). Comparative histological aspect of fibrotic areas before lipofilling (**A**–**D**) and after lipofilling (**E**–**H**). Low-magnification overview of pre-lipofilling fibrotic tissue adjacent to adipose tissue. The pre-lipofilling specimen shows compact fibrotic tissue characterized by dense collagen deposition and dilated intrafibrotic vascular structures. Adjacent adipose tissue displays morphological alterations consistent with prior radiotherapy. The compact fibrotic area (**A**) The pre-lipofilling specimen shows compact fibrotic tissue characterized by dense collagen deposition and dilated intrafibrotic vascular structures. Adjacent adipose tissue displays morphological alterations consistent with prior radiotherapy. The compact fibrotic area (**B**) shows low cellularity, confirmed in higher magnification images (**C**,**D**). Notably, the perifibrotic adipose tissue lacks active cellular infiltration. In the post-lipofilling biopsy (**E**), newly formed adipose tissue is present and demonstrates different stages of development (**F**). The newly formed connective tissue shows variable cellularity (**G**). Areas of immature connective tissue present increased cellular density, whereas more mature connective tissue demonstrates reduced cellularity as collagen fibers progressively form and organize. Marked heterogeneity of adipose tissue remodeling is observed (**H**). Early stages are characterized by hypercellular adipose tissue with fine collagen fibrils located between adipocytes. Later stages show a decrease in cellularity associated with the presence of collagen fibers organized into bundles oriented in different directions. These findings suggest a progressive and heterogeneous remodeling process of the grafted adipose tissue.

**Figure 6:**
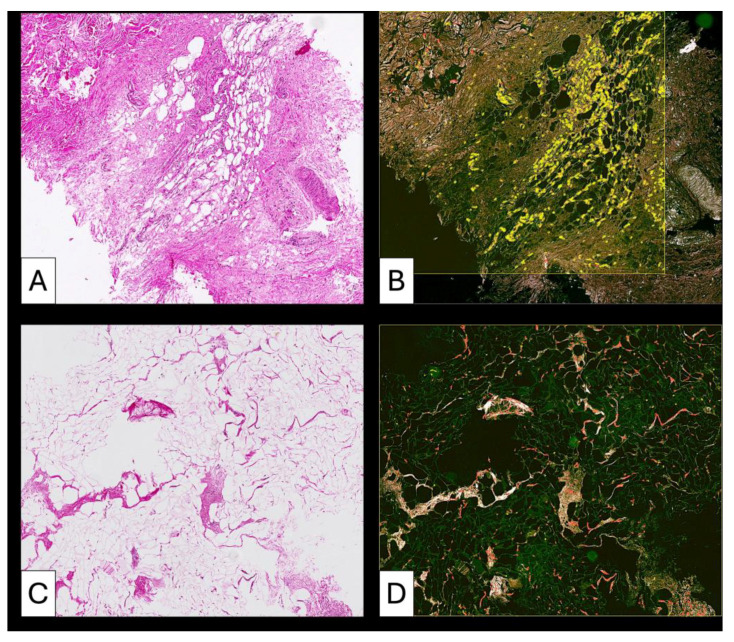
Pre-lipofilling fibrotic architecture in patient P3 (**A**–**D**). The pre-lipofilling specimen shows dense fibrotic tissue with a compact collagen structure and dilated vascular elements located within the fibrotic stroma (**A**,**B**). The fibrotic region displays low cellularity, as confirmed in higher magnification images (**C**,**D**). The adjacent adipose tissue shows morphological changes compatible with post-radiotherapy alterations and lacks signs of active cellular remodeling.

**Figure 7:**
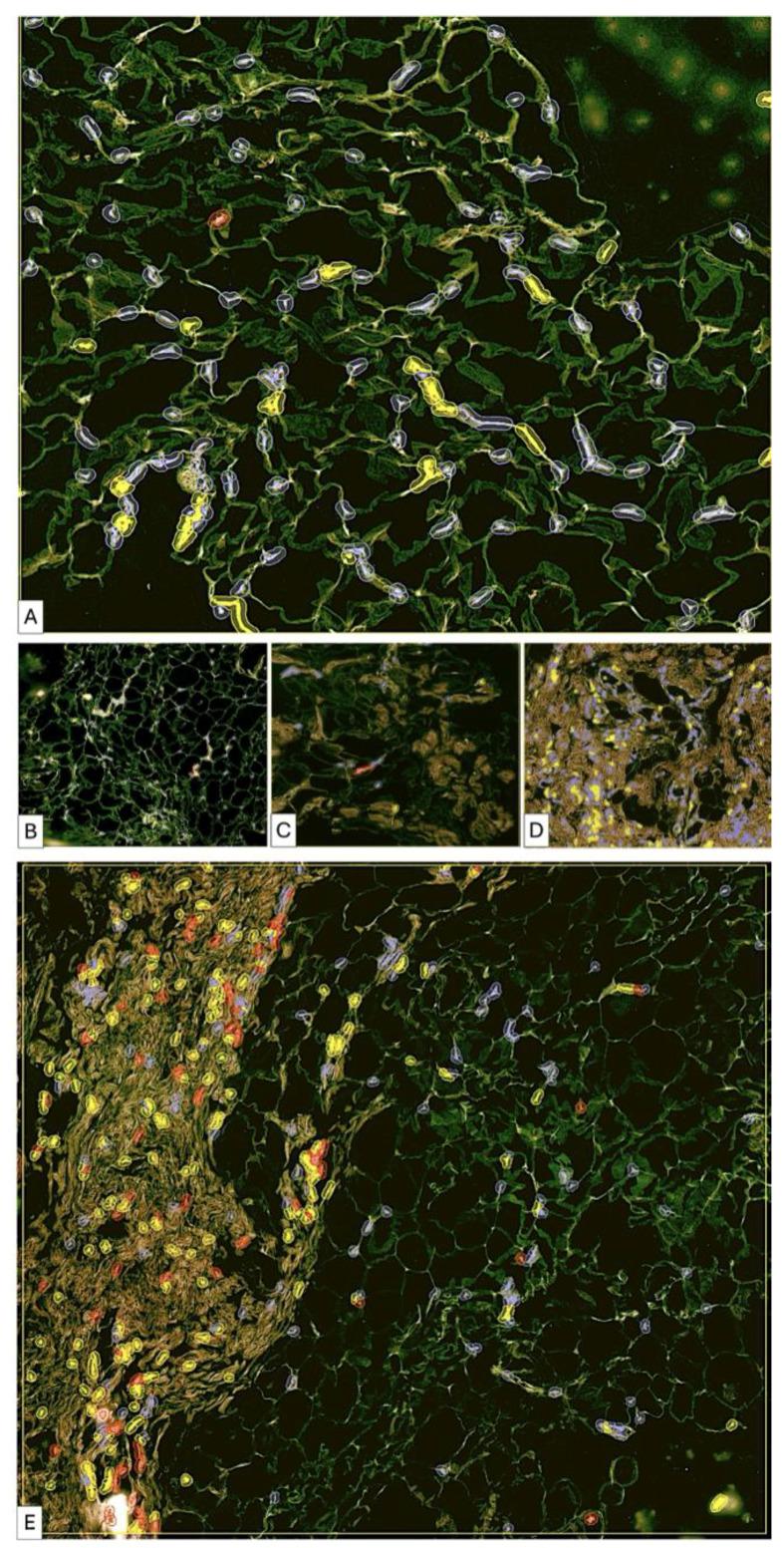
After lipofilling, adipose tissue remodeling demonstrates a heterogeneous pattern. Early stages show hypercellular adipose tissue with increased stromal cellularity (**A**). Fine collagen fibrils are present between adipocytes (**B**), indicating early extracellular matrix deposition. Areas with partial collagen organization and reduced cellularity are observed (**C**). Regions of dense irregular connective tissue containing isolated adipose islands with increased cellularity are also identified (**D**). In other regions, cellularity decreases as collagen fibers organize into bundles arranged in multiple directions. Areas of dense irregular connective tissue contain isolated adipose islands with increased cellularity, suggesting a stepwise or wave-like remodeling process. Comparative areas also highlight the coexistence of highly cellular adipose tissue and collagen-rich regions (**E**), emphasizing the heterogeneity of the remodeling process. Persistent post-radiotherapy tissue changes may contribute to delayed adipose remodeling, particularly in areas with persistent inflammatory alterations.

#### 3.3.4. Patient 4 (Figure 8 and Figure 9)

Baseline biopsy showed moderate inflammatory infiltrate, increased vascularity, and degenerative adipocyte changes. Post-lipofilling samples demonstrated alternating areas of organized adipose tissue and regions of collagen-rich connective tissue with well-structured collagen bundles. No significant inflammatory infiltrate was identified post-treatment.

**Figure 8:**
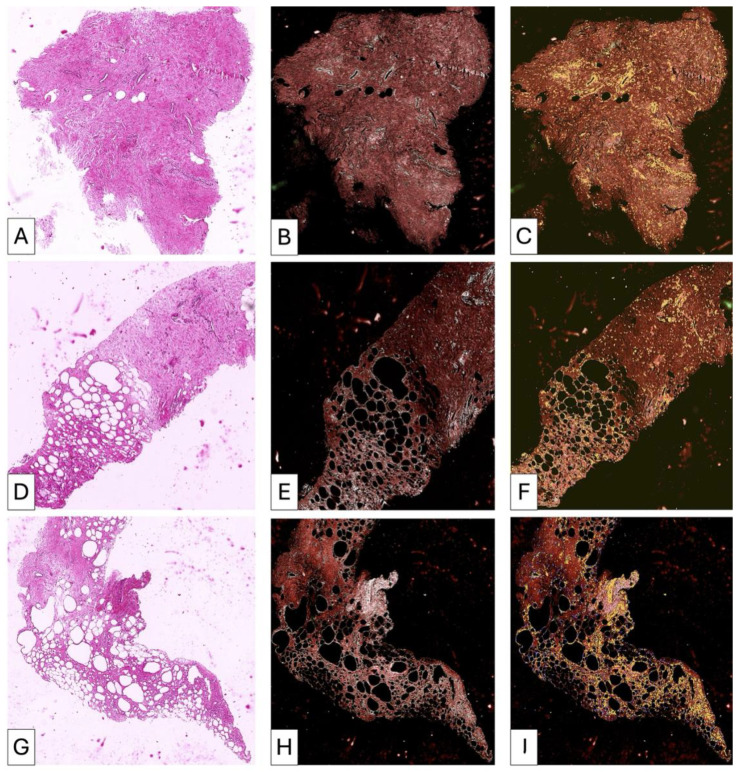
Samples of fibrotic tissue (**A**) and fibrotic plus postirradiated adipose tissue areas (**D**,**G**) from patient number 1. Compact fibrotic areas show isolated trapped adipocytes (**A**). Collagen-dense fibrotic tissue with compact organization is highlighted (**B**), with enhanced visualization of collagen distribution and structural density (**C**). In other regions, adipose tissue and fibrosis are closely interrelated (**D**,**G**). Highly vascularized fibrotic areas are observed (**D**). Adipose tissue with increased collagen deposition between adipocytes is shown (**E**), with corresponding visualization of collagen-rich areas (**F**). Additional areas demonstrate predominance of immature collagen fibers and increased cellularity (**H**), with spatial distribution of collagen fibers within adipose structures (**I**). These findings indicate advanced structural changes and ongoing remodeling processes in irradiated mammary tissue before lipofilling.

**Figure 9:**
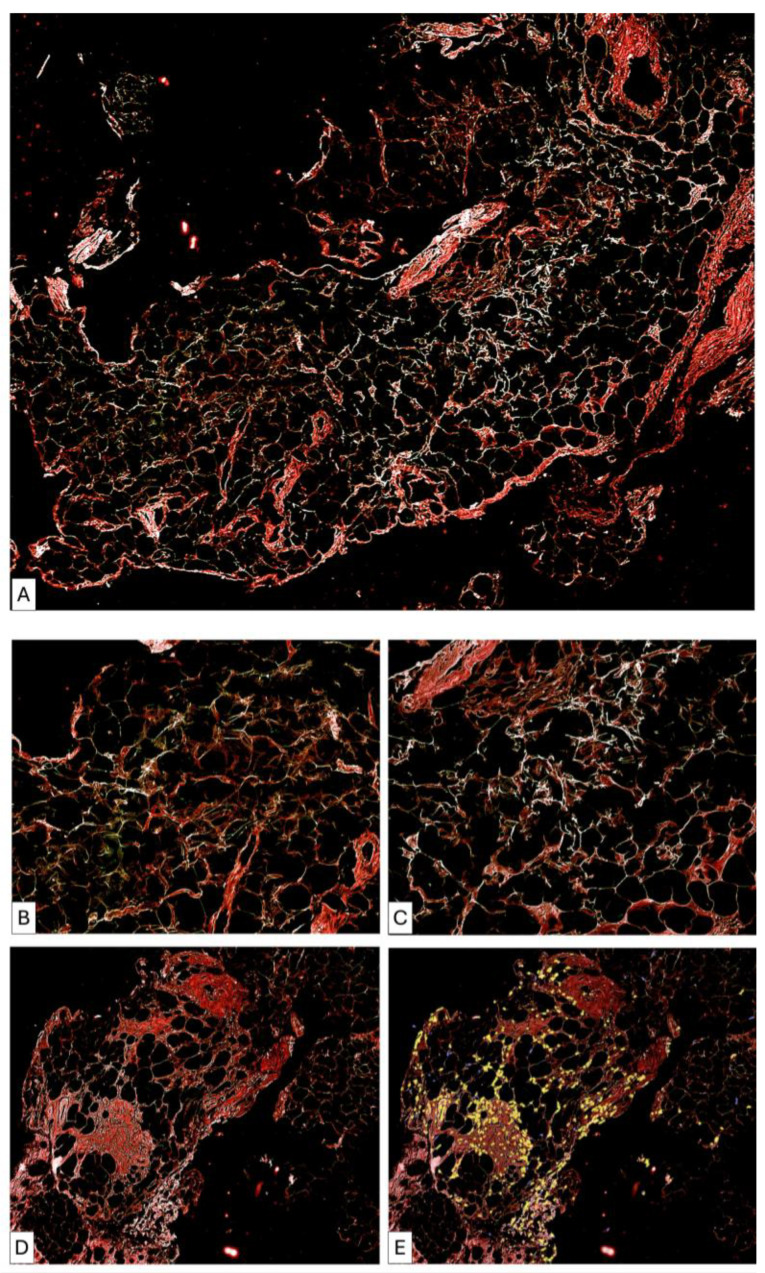
Post-lipofilling adipose tissue (**A**) showed a global disorganization microscopically detected by the presence of broken adipose cells (irregular green lines) and by an increased density in collagen fibrils. The collagen fibrils have tendency to form thicker fascicles but these are very rare and with patchy distribution in some areas. The collagen fibrils tend to form a loose network similar to that from the loose connective tissue (**B**) instead of dense irregular connective tissue but in other areas collagen fibrils (white) are short and not connected to each other (**C**). Structural heterogeneity from post-lipofilling areas is also characterized by the presence of patches of dense regular connective fibers arrangements (**D**) but highly cellular (**E**) which may suggest an ineffective and improper dense irregular connective tissue formation.

#### 3.3.5. Patient 5 (Figure 10)

Pre-treatment findings included vascular proliferation, perivascular collagen thickening, muscular fiber disruption, and moderate inflammatory infiltrate. Post-lipofilling biopsy revealed heterogeneous remodeling characterized by preserved adipose areas adjacent to regions of dense connective tissue organization. Focal pseudofollicular inflammatory aggregates were observed.

**Figure 10:**
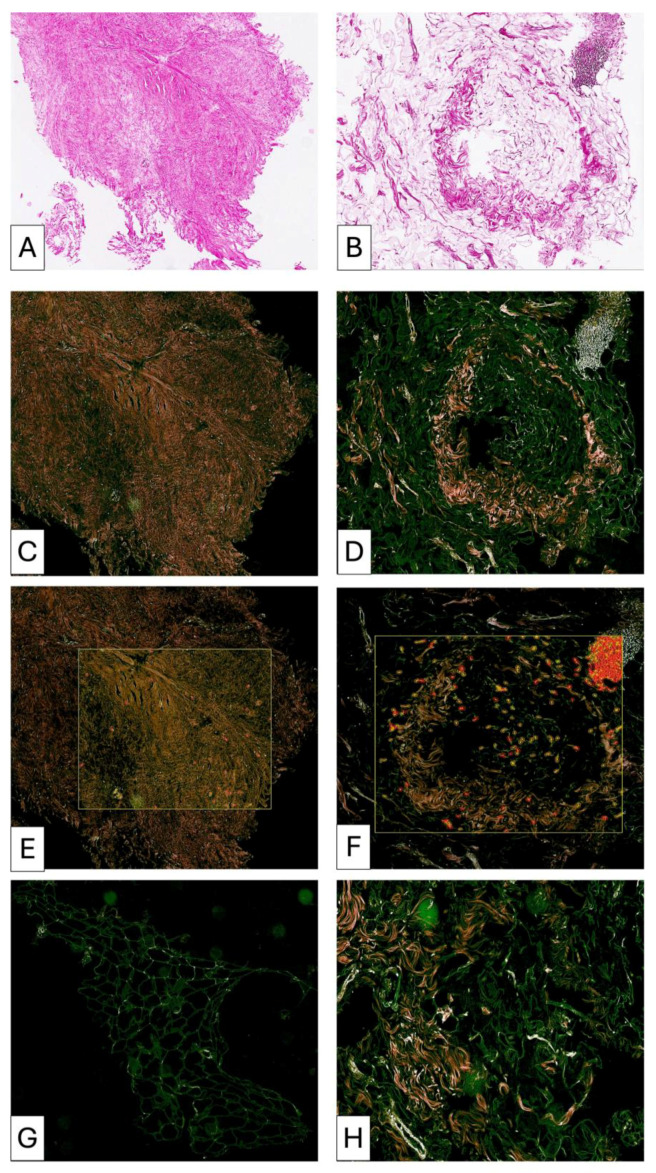
P5—Comparative histopathological evaluation of fibrosis and adipose tissue remodeling in patient P5 (**A**–**H**). (**A**) Pre-lipofilling fibrotic tissue characterized by dense connective stroma with collagen fibers predominantly arranged in parallel bundles, a pattern typical for mature fibrosis. (**B**) Post-lipofilling adipose tissue remodeling characterized by collagen fibers of variable length and thickness. Some fibers show a parallel orientation, but their density and organization differ from those observed in fibrotic tissue. Many collagen fibers present a multidirectional orientation, reflecting an active remodeling process. (**C**) RGB digital analysis highlights mature collagen fibers within the pre-lipofilling fibrotic area. These fibers appear predominantly brown, indicating compact and well-organized collagen bundles. (**D**) In contrast, collagen fibers identified in post-lipofilling areas show heterogeneous staining patterns. Young fibrillar collagen appears as fine linear white structures, representing early stages of collagen deposition before organization into mature bundles. (**E**) The fibrotic areas demonstrate extremely low cellularity. (**F**) In comparison, the adipose remodeling zones show high cellular density. Most cells are closely associated with collagen fibers, suggesting active extracellular matrix remodeling. (**G**) Native adipose tissue used for lipofilling shows normal architecture and appears green in RGB analysis, consistent with preserved adipose structure. (**H**) Adipose tissue undergoing remodeling shows partial preservation of adipose characteristics but with marked cellular disorganization. Numerous collagen fibrils in different stages of maturation are interspersed within the remodeling adipose tissue.

#### 3.3.6. Overall Semi-Quantitative Histological Overview

To facilitate structured comparison across all patients and timepoints, key histological parameters were summarized using a semi-quantitative descriptive grading system (0–3), as presented in Table 3.

### 3.4. Overall Remodeling Pattern

Across all five cases, adipose tissue remodeling following lipofilling demonstrated an individualized pattern. Structural transformation appeared heterogeneous and multi-stage, with coexistence of preserved adipose tissue, immature connective tissue formation, and areas of dense collagen organization.

The relative proportion between residual adipose tissue and dense disorganized connective tissue varied among patients.

**Table 1:** Patient and Procedural Characteristics.

Patient	Age (Years)	Type of Mastectomy	Time Since Radiotherapy (Months)	Interval Between Baseline and Post-Lipofilling Biopsy (Months)	Injected Fat Volume (mL)	Adverse Effects/Complications	Relevant Notes
P1	56	Radical	45	4.5	150	Deceased (unrelated to lipofilling)	—
P2	52	Radical	17	3	150	—	—
P3	40	Radical	6	4.4	120	—	Underwent DIEP reconstruction
P4	47	Radical	15	12	150	—	Tissue expander in place; capsular contracture; underwent DIEP reconstruction
P5	59	Radical	18	4	250	—	—

**Table 2:** Continuation of Table 1 presenting additional clinical, oncological, and treatment-related characteristics of the included patients.

Patient	Hypodermal Thickness Gain (mm)	Fascial Plane Definition	Neoadjuvant Treatment	Genetic Testing	Immunohistochemistry (IHC)	Relevant Personal History	Height/Weight	Radiotherapy	Histopathological Diagnosis
P1	5.5	Clear	Doxorubicin; Docetaxel; Epirubicin	—	ER 100% (3+); PR 100% (3+); HER2 negative; Ki-67 5%	Smoking history 25 years	162 cm/84 kg	25 sessions × 2 Gy = 50 Gy	Invasive breast carcinoma, no special type (NST), grade 2 (Nottingham score 7: 3 + 2 + 2)
P2	6	Clear	4 × EC dose-dense + 4 × Taxanes dose-dense	—	ER 98%; PR 98%; HER2 0; Ki-67 35%	—	160 cm/70 kg	25 sessions × 2 Gy = 50 Gy	Moderately differentiated invasive breast carcinoma G2 (possible lobular type)
P3	5	Partial	4 × EC + 4 × PTX + Pertuzumab + Trastuzumab	BRCA negative	ER 40%; PR 50%; HER2 3+; Ki-67 52%	-	160 cm/67 kg	25 sessions × 2 Gy = 50 Gy	Invasive breast carcinoma NST, G2, with mucinous component. Nottingham score 7 (3 + 2 + 2)
P4	8.4	Clear	4 × EC dose-dense; 4 × Paclitaxel	125-gene panel negative	ER 100%; PR 100%; HER2/neu 1+ Ki-67 61%	Superficial venous thrombosis, right upper limb	166 cm/66 kg	25 sessions × 2 Gy = 50 Gy	Invasive breast carcinoma NST, G2, Nottingham score 6 (2 + 2 + 2)
P5	4.2	Clear	4 × EC + 12 × Paclitaxel weekly	Negative	ER 90%; PR 10%; HER2 0; Ki-67 25%	—	165 cm/69 kg	25 sessions × 2 Gy = 60 Gy/30 fractions + 10 Gy boost	Lobular carcinoma G2

**Table 3:** Semi-quantitative descriptive grading of histological features in paired pre- and post-lipofilling specimens. Scale: 0 = none; 1 = mild; 2 = moderate; 3 = marked. Grading was derived directly from descriptive terminology in the original pathology report and applied uniformly across patients and timepoints.

Patient	Timepoint	Fibrosis (0–3)	Inflammatory Infiltrate (0–3)	Vascular Alterations (0–3)	Perivascular Thickening (0–3)
P1	Pre-lipofilling	3	3	3	2
	Post-lipofilling	1	3	3	1
P2	Pre-lipofilling	2	2	2	0
	Post-lipofilling	1	0	3	0
P3	Pre-lipofilling	2	0	0	0
	Post-lipofilling	2	0	1	0
P4	Pre-lipofilling	3	2	3	0
	Post-lipofilling	3	0	0	0
P5	Pre-lipofilling	3	2	3	3
	Post-lipofilling	3	1	1	1

## 4. Discussion

The present paired intra-patient histological analysis demonstrated that irradiated postmastectomy breast tissue exhibits a consistent baseline pattern of radiation-induced fibrosis, while post-lipofilling specimens reveal structurally demonstrable but heterogeneous remodeling. All pre-treatment biopsies showed collagen condensation, stromal disorganization, vascular alterations, and variable inflammatory activity, confirming the presence of an established fibrotic microenvironment prior to intervention. Following autologous fat grafting, structural changes were observed in all cases, including altered adipocyte architecture, interstitial collagen redistribution, and variable connective tissue reorganization. Importantly, remodeling was not uniform across patients, with patterns ranging from preserved adipose architecture to focal adipocyte loss and collagen-dominant transformation. These findings indicate that autologous fat grafting is associated with adaptive structural modification of irradiated tissue rather than complete reversal of fibrosis. Case-specific variability ranged from persistent inflammatory microenvironment in one patient to minimal inflammatory activity and predominant structural reorganization in others.

The baseline histological profile observed across all patients aligns with the established pathological spectrum of radiation-induced fibrosis. Chronic collagen condensation, stromal disorganization, and microvascular alterations are recognized hallmarks of irradiated soft tissue and reflect sustained fibroblast activation and extracellular matrix dysregulation following radiotherapy exposure [2,3,4,11]. Persistent TGF-β signaling, fibroblast-to-myofibroblast transition, and progressive matrix deposition have been described as central mechanisms underlying architectural stiffening and vascular compromise in irradiated tissues [2,3,4]. Histopathological analyses of irradiated breast skin further confirm the coexistence of dense fibrotic areas and residual adipose structures, highlighting the spatial heterogeneity characteristic of chronic radiation injury [12]. The uniform presence of these alterations in our cohort confirms that the sampled tissue represented an established fibrotic microenvironment prior to intervention.

Autologous fat grafting has been increasingly employed in irradiated breast tissues to improve tissue pliability, reduce fibrosis-related stiffness, and facilitate subsequent reconstructive procedures. Recent reconstructive approaches combining fat grafting with flap-based techniques have further supported its role in improving outcomes in irradiated breast tissue [13]. Early clinical observations suggested that lipoaspirate transplantation may partially reverse radiation-associated tissue damage through regenerative mechanisms [6]. Subsequent clinical series in postmastectomy irradiated chest wall reconstruction demonstrated improved soft-tissue quality and enhanced suitability for implant-based reconstruction following staged lipofilling [14,15]. Similar long-term clinical improvements in tissue quality and esthetic outcomes following structured fat grafting protocols have also been reported [16]. Systematic reviews and narrative analyses further support the beneficial role of autologous fat transfer in radiation-induced soft-tissue injury, although the majority of reported outcomes remain clinical or imaging-based rather than histologically documented [7,17]. In this context, direct human intra-patient histological comparisons remain scarce. The present paired-biopsy design therefore extends existing literature by providing structural documentation of stromal reorganization within an established irradiated microenvironment.

The structural changes observed following lipofilling are biologically coherent with the proposed regenerative properties of adipose-derived cellular components. Adipose-derived stem cells (ADSCs) and stromal vascular fraction elements are known to secrete pro-angiogenic, anti-inflammatory, and matrix-modulating cytokines that may influence irradiated stromal microenvironments [8,18]. Experimental models have demonstrated improved vascular density, modulation of fibroblast activity, and partial reorganization of extracellular matrix architecture in irradiated tissues following fat grafting [19,20]. The focal collagen redistribution and areas of immature connective tissue formation observed in our specimens may therefore reflect an adaptive stromal response, rather than simple volumetric augmentation. Nevertheless, in the absence of immunohistochemical or molecular analyses, the present findings should be interpreted as structural documentation of remodeling, rather than direct evidence of specific cellular pathways.

The heterogeneous structural remodeling observed across patients suggests that adipose tissue integration in irradiated environments does not follow a uniform regenerative pathway but rather a spatially variable adaptation process influenced by the local inflammatory and fibrotic microenvironment.

The present findings suggest that adipose graft integration in irradiated tissue may occur through a staged remodeling process involving progressive collagen reorganization and variable adipose–connective tissue transformation rather than uniform tissue regeneration.

From a clinical perspective, the histological findings observed in the present study provide structural context to previously reported functional and imaging-based improvements following lipofilling in irradiated chest wall tissues. In prior analyses of the same prospective cohort, both symptomatic outcomes and high-resolution ultrasound assessments demonstrated improvement in tissue quality after autologous fat grafting [10,21]. Clinical benefits of autologous fat grafting in postmastectomy patients have also been reported in terms of symptom improvement, including reduction in postmastectomy pain syndrome [22]. The current paired histological evaluation complements those findings by documenting microscopic stromal reorganization within the irradiated recipient bed. Taken together, these multimodal observations suggest that structural adaptation following fat grafting may involve coordinated macroscopic and microscopic remodeling processes. This integrated interpretation supports the concept that autologous fat grafting may act, not solely as a volumetric adjunct, but as a biological modulator of irradiated tissue architecture.

A notable aspect of the present findings is the heterogeneous nature of the observed remodeling. Structural adaptation was not uniform across specimens, and residual fibrotic architecture persisted in several areas despite evidence of focal collagen redistribution and connective tissue reorganization. This variability likely reflects the intrinsically complex biology of radiation-induced fibrosis, in which chronic microvascular damage, differential fibroblast activation, and spatially variable extracellular matrix deposition coexist within the same tissue bed [4,11]. Moreover, individual differences in radiation dose distribution, timing from radiotherapy, and patient-specific regenerative capacity may further influence the extent of stromal responsiveness to fat grafting.

Several limitations should be acknowledged when interpreting the present findings. The sample size was limited, reflecting the invasive nature of paired intraoperative biopsies in irradiated tissue, and therefore restricts statistical generalizability. Histological evaluation was based on conventional hematoxylin–eosin staining without adjunct immunohistochemical or molecular analyses, precluding direct assessment of specific cellular pathways or cytokine activity. In addition, although sampling was performed from anatomically comparable regions, the intrinsic heterogeneity of radiation-induced fibrosis may introduce spatial variability between biopsy cores. Nevertheless, the prospective paired design and intra-patient comparison strengthen internal validity and provide structured morphological documentation within a clinically relevant reconstructive setting.

The present findings contribute to a growing body of evidence suggesting that autologous fat grafting may influence the structural characteristics of irradiated recipient tissues beyond simple volumetric augmentation. By documenting paired intra-patient histological changes within a clinically relevant reconstructive context, this study provides morphological support for the concept of stromal modulation in radiation-damaged breast tissue. Future investigations integrating immunohistochemical profiling, molecular pathway analysis, and larger prospective cohorts will be essential to further elucidate the biological mechanisms underlying this remodeling process. Within its current scope, the present work offers structured human histological evidence that supports a paradigm in which fat grafting may function as an adaptive modifier of the irradiated tissue microenvironment.

Although the cohort size precludes temporal correlation analysis, the variability in biopsy intervals suggests that structural remodeling is detectable as early as 3 months and may continue to evolve beyond 6–12 months. This observation supports the concept of a progressive, multi-stage tissue adaptation process following lipofilling.

The stepwise and patient-dependent nature of adipose-to-connective tissue transformation observed in our cohort may have practical implications for reconstructive timing. Given that dense disorganized connective tissue organization appears to evolve progressively after lipofilling, a longer interval between fat grafting and definitive reconstruction could allow more complete stromal stabilization in selected patients. However, this hypothesis requires validation in larger prospective studies.

Core needle biopsies sample a limited tissue volume and may not fully capture spatial heterogeneity of radiation-induced fibrosis.

In addition, the semi-quantitative scoring system used in this study was exploratory and observer-dependent, as it was not based on a previously validated histological scale.

An important limitation of this study is that histological evaluation was not performed in a blinded manner. This may have introduced observer bias in the interpretation of post-treatment changes.

Another important limitation of this study is the absence of a control group. No comparisons were performed with non-irradiated breast tissue or with irradiated tissue not treated with lipofilling. As a result, the observed histological changes cannot be definitively attributed to autologous fat grafting alone and may partially reflect the natural evolution of irradiated tissue over time.

The present study should be interpreted as a hypothesis-generating histological pilot investigation. The limited sample size reflects the invasive nature of paired intraoperative biopsies in irradiated tissue and restricts statistical generalizability. Consequently, the findings are intended to provide preliminary structural observations rather than definitive conclusions regarding the effects of autologous fat grafting.

## 5. Conclusions

This prospective paired-biopsy pilot study suggests that autologous fat grafting in irradiated postmastectomy breast tissue may be with structurally detectable remodeling at the histological level. Observed changes included heterogeneous reorganization of collagen architecture, focal insertion of collagen fibers between adipocytes, and variable preservation of adipose tissue within the treated area. In several cases, remodeling included progressive transformation of adipose tissue into dense disorganized connective tissue, occurring in a stepwise and patient-specific manner.

The post-treatment histological findings suggested structural patterns that differed from the initially anticipated uniform regenerative response, highlighting the complexity of tissue adaptation following lipofilling.

Radiation-induced baseline alterations were observed across all patients. Post-treatment remodeling patterns varied individually, reflecting patient-specific patterns of adipose–connective tissue transformation. The coexistence of preserved adipose regions and areas of connective tissue reorganization suggests the concept that tissue adaptation following lipofilling may represent a dynamic and spatially heterogeneous process rather than uniform regeneration. Remodeling appears detectable within three months and may continue to mature over longer intervals.

Although definitive mechanistic conclusions cannot be drawn based on hematoxylin and eosin staining alone, the present findings provide direct human histological documentation of structural changes occurring after autologous fat grafting in irradiated tissue.

Further studies incorporating quantitative histological scoring, immunohistochemical markers, and larger patient cohorts are required to better define the biological pathways underlying this remodeling process. These findings should be interpreted as preliminary observations derived from a hypothesis-generating pilot study.

## Data Availability

The data presented in this study are available on request from the corresponding author due to patient privacy and ethical restrictions.

## Abbreviations

The following abbreviations are used in this manuscript:

ADSCs	Adipose-Derived Stem Cells
ECM	Extracellular Matrix
H & E	Hematoxylin and Eosin
IHC	Immunohistochemistry
RGB	Red Green Blue
SVF	Stromal Vascular Fraction
NST	No Special Type
